# Christoph Nikendei, Till Johannes Bugaj, Anna Cranz, Alina Herrmann, Julia Tabatabai: Heidelberger Standards der Klimamedizin – Wissen und Handlungsstrategien für den klinischen Alltag und die medizinische Lehre im Klimawandel

**DOI:** 10.3205/zma001607

**Published:** 2023-05-15

**Authors:** Lorena Morschek

**Affiliations:** 1 Universitätsklinik Heidelberg, Klinik für Allgemeine Innere Medizin und Psychosomatik, Heidelberg, Germany

## Bibliographical details

Christoph Nikendei, Till Johannes Bugaj, Anna Cranz, Alina Herrmann, Julia Tabatabai

**Heidelberger Standards der Klimamedizin – Wissen und Handlungsstrategien für den klinischen Alltag und die medizinische Lehre im Klimawandel**

Publisher: Heidelberger Klinische Standards

Year of publication: 2023, 352 pages, price: € 34,99

ISBN: 978-3-00-074681-9

## Review

The climate crisis threatens our natural livelihoods and civilization in many ways. With the steady increase in mean global temperature, the crossing of irreversible climate tipping points is becoming increasingly likely. There is a threat of a massive acceleration of biodiversity loss, acidification of the oceans, more frequent food shortages, an increase in extreme weather events, and climate-related refugee movements.

As a compact “pocket book”, the *Heidelberg standards of climate medicine* provides broad knowledge, as well as practice-oriented recommendations for action on the scientific basics, the (medical) treatment of physical and psychological effects of the climate crisis, and the necessary transformation processes towards a more ecologically sustainable health sector. In 12 chapters with a total of 45 articles, the participating authors summarize the current state of research and information from their (medical) practice and provide useful tips and advice for the everyday professional life of all participants in the health sector.

First, the *Heidelberg standards of climate medicine* provide a brief and concise overview of the *scientific basics of the climate crisis* (chapter 1) and their *impact on natural and social systems* (chapter 2). The knowledge of these basics will, on the one hand, help the reader with regard to the following book chapters, and, on the other hand, it should serve as a practical tool in (scientific) discourse.

Two comprehensive chapters then highlight the numerous direct and indirect impacts of the climate crisis on human health. In the third chapter on the *physical impacts of the climate crisis*, it becomes evident how closely environmental changes (e.g., more frequent extreme weather events, heat waves, increased incidence of tropical pathogens) are linked to human health: The occurrence of many diseases, such as heat stroke, COPD, allergies, diabetes, or kidney failure, are influenced by the climate crisis. In addition to explaining this relationship, the chapter presents definitions relevant to clinicians, pathophysiological processes, key recommendations for clinical care, and helpful therapeutic strategies. The* psychological effects of climate change* are reflected upon in more detail in chapter 4, ranging from trauma in the context of natural disasters, to connections between climate, weather, and suicidality. Additionally, chapter 5 is dedicated to general *cognitive processes and biases in psychological perceptions and processing of climate change*. The discrepancy between knowledge and action regarding the climate crisis is also impressively illustrated.

Subsequently, the *Heidelberg standards of climate medicine* reveals concrete *fields of action in the health sector* (including the reduction of emissions by means of changing the food supply, energy management, and drug supply in hospitals) in order to decisively counter the health crisis stemming from the climate crisis (chapter 6). Afterwards, various *health co-benefits of climate protection measures* are explained using plausible examples (chapter 7), framed by *practical tips for climate-sensitive health counseling* and *patient-oriented climate communication* (chapter 8). Furthermore, it is presented in an understandable and precise way, how a *sustainable transformation of the health sector* can succeed concretely (chapter 9). Moreover, it is explained how findings on the climate crisis can be integrated into *research and teaching* in a targeted manner (chapters 10 and 11). Finally, considerations on the *medical ethos in times of climate crisis* are presented and various initiatives in the health sector are introduced (chapter 12). In this respect, the contentual focus of the book illustrates both the complex effects of the climate crisis, as well as the remaining scope of action, both on an individual and structural level. Numerous tables and illustrations provide a quick overview of key aspects of the chapters. A more in-depth discussion of the topics is made possible by the main text and a large number of (in part further) source references.

Reading the *Heidelberg standards of climate medicine* is recommended in particular for interested stakeholders in the health sector, who want to engage with the basics and consequences of the climate crisis for their everyday professional life, implement gained knowledge in their (medical) practice, research and teaching, and also want to understand and help shape transformation processes.

## Competing interests

The author declares that she has no competing interests.

